# Data science in clinical and translational research: Improving the health of the data to knowledge pipeline

**DOI:** 10.1017/cts.2020.569

**Published:** 2021-03-09

**Authors:** Christopher J. Lindsell, Gina-Maria Pomann, Robert A. Oster, Sean D. Mooney, Felicity T. Enders

**Affiliations:** 1 Department of Biostatistics, Vanderbilt University Medical Center, Nashville, TN, USA; 2 Department of Biostatistics and Bioinformatics, Duke University, Durham, NC, USA; 3 Division of Preventive Medicine, Department of Medicine, University of Alabama at Birmingham, Birmingham, AL, USA; 4 Department of Biomedical Informatics and Medical Education, University of Washington, Seattle, WA, USA; 5 Division of Biomedical Statistics and Informatics, Department of Health Sciences Research, Mayo Clinic College of Medicine and Science, Rochester, MN, USA

In this themed issue of the *Journal of Clinical and Translational Science*, our goal was to curate a set of manuscripts that would highlight current innovations in data science that are of relevance to clinical and translational researchers. We planned to cover methodological advances, education, and the systems and processes by which data scientists contribute to clinical and translational research. Our open call for manuscripts led to a rather diverse array of submissions that truly reflects the breadth and depth of data science in clinical and translational research. Given such breadth and depth, we were hard pressed to answer the question of “what fits?”

Our definitions of data science started to expand uncontrollably as we considered a diverse set of statistical and computational methods, data collection, data consumption, data analysis, data processing, data governance, and more. We explored the many different facets of data science, from theoretical model development, to the pipelines that move data from an electronic health record into a structured dataset for analysis, to the core competencies of a data scientist. Ultimately, we concluded that defining data science is somewhat similar to defining medicine. While the concept might be well understood by the lay person, and much can be done by a patient to practice healthy behaviors and self-care, the subtleties and nuances start to come into focus as problems become tougher, needs become more specialized, and rare or unusual problems are in need of resolution. We know that doctors with specific expertise and specialties make different contributions and serve different needs, and the application of data science in clinical and translational research is analogous.

Take a collaborative biomedical informatician or biostatistician as an example. Such a data scientist might serve on the front lines, meeting with investigators, administrators, clinicians, and data scientists every day to navigate through a complex collaborative process that includes everything from hypothesis generation to regulatory controls on data management systems. The core driver of daily work is to correctly answer research questions by optimizing the quality of the data being collected and the rigor and reproducibility with which those data are analyzed. The collaborative data scientist tends to focus more on the general application of methodological principles than on specialized analytical or computational problems. The generalist might be able to conceptualize the data pipelines, understand the difference between conditional and marginal models, recognize clustering and identify when clustered methods are required, and have some familiarity with OMOP, C-DISC, FHIR, ICD-10, and many of the other data and data transport standards. Even when the generalist has an area of independent specialization, he or she is likely to hit points in the collaboration when a different specialty is required. For example, the generalist may not have the extensive expertise needed to write the code that shifts large volumes of data from the electronic health record and manipulates it into an accessible structure. In other cases, a specialist may need to be enlisted to properly develop an unbiased approach to estimating the 95% confidence intervals for a proportional odds model with both fixed and random effects, or developing machine learning algorithms that overcome the constraints of uncertainty in feature inputs. Conversely, there are numerous aspects of the research pipeline that require data scientists with a deep understanding of the details of the clinical or scientific area and the methodological techniques needed to solve problems, but such data scientists must also have some broad knowledge of general principles and best practices so that fundamental mistakes are avoided.

To us, this sounds a lot like medicine. While a general practitioner may perform the initial evaluation of a patient and provide maintenance healthcare, at some point they may need to refer the patient to a specialist. In clinical research, the investigator might bring the research question to a generalist, who can explore all of the possible things that could undermine the project’s health – including whether the investigator is over-reliant on Dr. Google and has preconceived notions of how to approach the problem, and what the solution is. The generalist must attempt to make a diagnosis. Once a diagnosis is made, simple things might be adequately treated with a *t*-test (analogous to statins in a patient with hyperlipidemia). More complex data or diagnostic conundrums might be referred to a specialist. Simple things that violate assumptions might also need to be referred, as might occur for the patient with high cholesterol whose liver function is adversely affected by the statin.

Also paralleled in medicine, the solution to any problem will be guided by the experience set and training of both investigator and data scientist, and the relationship between them. An investigator collaborating with a generalist might have consistent and well considered diagnostics, a series of preventive measures (e.g. training on contemporary data science methods), and access to a network of specialists when needed. An investigator whose research focus is on discovery of the genetic basis of disease might have a close collaboration with a statistical geneticist or a bioinformatician, just as a patient with heart failure might have a close relationship with his or her cardiologist. In this case, we hope that if the patient asks the cardiologist about arthritis, a referral to a rheumatologist occurs or the primary care provider is re-engaged (although we note that a patient consulting a surgeon might find themselves going under the knife more frequently than the patient consulting with an internist, which might or might not be entirely appropriate).

As we carried on building out this analogy, it became clear we could take it to an extreme – perhaps we already have. Nonetheless, we found that every article in this special issue fits. Each one elucidates the many general and special ways that data science supports the strength and health of clinical and translational research. For example, a structural similarity between medicine and data science is the array of ancillary professionals who are integral to the successful care of a patient. In data science, these include data managers, technical support, and programmers, as well as others on the research team such as study coordinators, research technicians, and clinical and translational science investigators. In “Eight Practices for Data Management to Enable Team Data Science,” McDavid *et al.* have organized and refined the process of data collection with the open source LabKey platform [1]. One of their goals was to achieve data collection that integrates the full collaborative research team, from study coordinator to data scientist, in a single platform, thereby increasing understanding throughout the team and minimizing time and errors. In our medical analogy, this is much like using an electronic health record to provide the same insights and data on the patient to the entire medical team (and beyond), allowing all to engage with the patient and with one another using a common set of critical information and tools.

The availability of data generated by teams of professionals across many care settings in the electronic health record and in the operational, administrative and financial systems of a health care organization provides an incredibly rich environment for discovery through data science. Arbet *et al.* discuss some of the challenges of using machine learning to parse large datasets from this rich environment. In “Lessons and Tips for Designing a Machine Learning Study Using EHR Data,” they argue that the black box nature of many machine learning algorithms mixed with the lure of simple interfaces to methodologically intensive tools has led to the misconception that machine learning alone can overcome issues with data within large databases [2]. In our medical analogy, this is akin to suggesting that simply seeing output from a full body scan can fully replace interpreting a targeted diagnostic test applied in the context of a patient’s medical history. There is a reason physicians place strong reliance upon a full medical history; it provides background and context for what is detected in the patient, informing the critical pretest probability that will drive meaning for many test results. In the realm of data science, background knowledge of the strengths and weaknesses inherent in the data based upon its provenance act as a kind of pretest probability, providing insights into which models or methods are reasonable and the strength of the resulting evidence. As such, Arbet *et al*. recommend consideration of the data and analytic design in machine learning studies, which we expect will increase the impact of quality data science.

One major consideration when handling data is how to treat missingness. This is particularly important in observational studies, such as when using electronic health record data. For studies that have a time-to-event outcome, Solomon *et al.* assess different methods for imputing missing data [3]. In their “Comparison of Regression Imputation Methods of Baseline Covariates that Predict Survival Outcomes,” they conclude that two contemporary methods outperform traditional statistical methods of the past. This emphasizes that it takes data scientists with specific expertise to develop new techniques to solve precise problems, just as a surgeon might develop a new technique to revolutionize a procedure. For new techniques to be of benefit beyond the individual use case, they must be disseminated to and accessible by generalists. This requires the generalist to stay up to date as best practices evolve. Continuing medical education is a requirement for medical practice; the same standard might be considered appropriate for data scientists. Over time, we expect that a more clear differentiation of specialist skills will emerge within data science, as will the expectation that the data scientist has core competencies and continuing education.

After completing a graduate degree, it is rare for an individual to have sufficient depth within specialty areas to act as either a generalist or a specialist in data science. It is clearly critical for data scientists to gain increasing expertise over time. In “Methods for Training Collaborative Biostatisticians,” Pomann *et al.* describe a framework for developing and maintaining technical, professional and communication skills for data scientists, including training in the clinical or scientific topic domain [4]. This formalization of data science skill development and maintenance is analogous to initial training of medical trainees and the continuing medical education framework. Training in data science should not end with the data scientist, though. Just as the physician might teach a patient to inject their own medications, or to take home blood pressure readings, there are many opportunities when the clinical and translational researcher can do their own data collection, management, and analyses. The key, in our opinion, is to know the limits of knowledge and to get specialist help when needed. This is one finding described in “Learning Gaps among Statistical Competencies for Clinical and Translational Science Learners” by Oster *et al.*[5] They describe the statistical topics in data science that might be done by an investigator, and highlight that training on limitations is essential to ensuring that data are not collected with bias or error, not summarized with information loss, and not analyzed in violation of applicable assumptions.

Every one of the manuscripts in this issue illustrates one or more aspects of how data science can improve the health of clinical and translational research. We are encouraged at the increasing integration of data science into clinical research, the growing demand for transparency and purpose behind clinical algorithms, and the increasing expectations of rigor and reproducibility of the data pipelines. We also hope that the parallel of medicine helps to illuminate the breadth and depth of data science. The next time someone asks what data science is, rather than rely on exclusionary definitions and elitist perspectives, consider the medical analogy. If someone is ensuring the health of the data to knowledge pipeline, then perhaps they are doing data science.
